# Feasibility, acceptability, and perceived benefits of a creative arts intervention for elementary school children living with speech, language and communication disorders

**DOI:** 10.3389/frcha.2024.1322860

**Published:** 2024-06-05

**Authors:** T. Léger-Goodes, C. M. Herba, Z. Moula, A. Mendrek, K. Hurtubise, J. Piché, M. Gilbert, M. Bernier, K. Simons, N. Bélanger, J. Smith, C. Malboeuf-Hurtubise

**Affiliations:** ^1^ Department of Psychology, Université du Québec à Montréal, Montreal, QC, Canada; ^2^ Research Centre of the CHU Sainte-Justine, Montreal, QC, Canada; ^3^ Department of Care in Long-Term Conditions, King’s College London, London, United Kingdom; ^4^ Department of Psychology, Bishop’s University, Sherbrooke, QC, Canada; ^5^ Research Centre of the CHUS, Sherbrooke, QC, Canada; ^6^ Faculty of Health Sciences, McMaster University, Hamilton, ON, Canada; ^7^ Department of Preschool and Primary Education, Université de Sherbrooke, Sherbrooke, QC, Canada

**Keywords:** art-based intervention, school-based intervention, children's mental health, speech, language, communication difficulties (SLCD), therapeutic arts, self-determination theory (SDT)

## Abstract

**Background:**

Children with speech, language, and communication disorders require specialized support in response to their emotional expression challenges. Not only is such support key for their development, but it is also essential for their mental well-being. Art making emerges as a valuable tool for enabling these children to convey emotions both verbally and non-verbally, fostering a positive self-concept. School-based arts interventions have the potential to improve self-concept and emotional expression, and more generally, the quality of life. However, there is limited empirical evidence regarding the feasibility, acceptability, and perceived effectiveness of a manualized, school-based creative arts intervention for this specific group.

**Aim:**

This study aimed to develop and evaluate the feasibility, acceptability, and perceived benefits of an art-based intervention for elementary school students with speech, language, and communication disorders, using qualitative methods to obtain diverse perspectives.

**Results:**

The findings indicate that the intervention was feasible and well received, as reported by teachers, students, and facilitators. Participants also discussed potential positive outcomes, including emotional expression, emotional growth, and fulfilment of autonomy, competence, and relatedness needs. Students found the workshops conducive to sharing emotions and took pride in the creative process. Teachers gained deeper insights into their students, fostering positive classroom relationships. Observational data triangulated these findings.

**Conclusion:**

This innovative project suggests that art-based interventions can potentially benefit students’ emotional expression, but further experimental studies are needed to substantiate these effects.

## 1 Introduction

Children with speech, language and/or communication disorders (SLCD), an umbrella term for the various problems in the realm of communication, experience difficulties in receptive and expressive language and the social use of language. The American Speech-Language-Hearing Association defines speech disorders as impairments in creating speech sounds, including articulation and fluency (1). Language disorders include problems in comprehension (receptive; understanding others) or use of spoken and/or written information (expressive; conveying a message). Communication disorders include speech disorders, language disorders, as well as hearing disorders (1). The prevalence of SLCD ranges from 9% to 13% in the general population (2, 3). These difficulties are often comorbid with various mental health and neurodevelopmental diagnoses, including autism spectrum disorder (ASD), attention deficit and/or hyperactivity disorder (ADHD), depression, anxiety, as well as other social and behavioral difficulties, such as aggressive behaviors (4–7). Although interventions that target everyday language and communication skills are essential to support children with SLCD (8, 9), it is also important to simultaneously provide mental health support to enable emotional awareness and expression, as these children often suffer psychological consequences of their communication difficulties (6). However, relying on typical verbal therapies can undermine the advantages of such mental health interventions with this population (10). Mental health professionals who work with children with SLCD identified that this population needs specific adaptation of typical talking therapies like cognitive-behavioral therapy (CBT), as they may have difficulties in conversation, language interpretation, and expression of their thoughts and emotions (11). For example, adapting CBT to include a focus on bodily sensations and other substitutions to talking are strongly advocated for by these therapists (11). Hence, approaches using the arts as an alternative way to promote emotional awareness and expression may be especially beneficial for children with SLCD (12).

### 1.1 SLCD and self-determination theory

Although children with SLCD are more at risk of developing various mental health problems (13), very few interventions specifically target mental health symptoms like anxiety or depression. To date, research including students with various learning disabilities and SLCD has focused on supporting children's basic psychological needs for autonomy, competence and affiliation to foster well-being in many different life domains (14–17). Self-determination theory, a macro theory of human motivation, posits that supporting these universal psychological needs is essential for a flourishing life (18). The need for autonomy can be defined as the desire to experience a sense of volition and choice. It involves feeling that one's own behavior and action is self-directed and congruent with personal values and interests. The need for competence consists of seeking to be effective and capable in handling challenges and achieving desired outcomes. This includes mastering skills and accomplishing meaningful tasks. Finally, the need for relatedness refers to the desire to connect with others, experience meaningful social relationships, and feel a sense of belonging within a social context. Creating spaces that support these three psychological needs can foster children's creativity, motivation, and school engagement (18). Children with SLCD could particularly benefit from interventions that support their autonomy, competence, and relatedness, as they face additional challenges in these domains (19). Indeed, autonomy, supportive experiences, feeling connected to peers, and tasks that enable a sense of competence can all contribute to emotion expression through introspection and self-understanding (20). Art-based interventions may be particularly beneficial for introspection and emotional expression in youth who may not possess the vocabulary to thoroughly describe their emotions (21). As such, these interventions can facilitate communication for children who experience trouble verbally communicating their thoughts and feelings, which could, in turn, foster feelings of competence (21, 22). Furthermore, the focus on subjective experiences and the creative process, rather than the finished piece of art, makes such interventions particularly suitable for children with SLCD. However, to date, no research has evaluated whether art-based interventions can effectively promote emotional expression and self-determination in children, namely those with SLCD.

### 1.2 Art-based interventions

Art-based approaches may be particularly beneficial for children and adolescents. Traditional therapeutic methods like individual psychotherapy have become increasingly inaccessible, and wait-lists times are at an all-time high (23, 24). Hence, art-based approaches could serve as an effective alternative for youth, particularly as they provide a sense of empowerment and prompt participants to share their work, thoughts, and feelings with their peers (25, 26). In youth populations, art-based interventions have namely served to overcome resistance to therapy (21). Furthermore, the arts (e.g., painting, sculpture, dance, etc.) allow psychologists to adapt their interventions according to the demographic they are working with. This capacity to adjust an approach is crucial to create interventions that align with the context of their participants, such as offering age and culturally appropriate activities (27, 28). Hence, art-based interventions have a promising potential to allow for introspection and emotional expression in children, supporting their mental health.

School settings may be particularly suitable for implementing art-based interventions. Indeed, in a review of eight studies involving school-based arts interventions among children aged 5–12 years old, Moula (29) found that integrating creative arts into the classroom had significant positive effects in improving children's quality of life, anxiety, self-concept, problem-solving skills, attitudes towards school, as well as emotional and behavioral difficulties. Another review of the literature on school-based art therapy found that creativity, expression of emotions through art, and finding meaning within the artwork could lead to better classroom behavior, improved self-concept, and help with separation anxiety disorder in children (30). However, only four experimental studies met the inclusion criteria for the review and the authors highlight that further evidence is needed to support these findings. Although few studies have examined the potential mental health outcomes of school-based art interventions, initial evidence from the present research team supports that art therapy can improve children's well-being and reduce psychological distress (31). Moreover, school-based drama therapy for children with autism and/or various developmental disorders has been linked to improved confidence in expressing emotional needs, increased use of creativity and imagination, and improved working memory (32). Other effective art-based interventions in schools include storytelling, drawing, puppetry, songwriting, and activities based on empowerment, such as creating characters based on children's *superpowers* (33). Noted benefits from these creative activities include children's increased sense of personal strength, confidence, identity, and positive emotions (33). Art-based interventions can also extend therapeutic relationships when conducted in a group setting, particularly when creative and interpretive processes are combined (25). Indeed, group settings can provide additional value to art-based interventions through peer support, self-regulation, and cooperation (34). School-based art interventions, when conducted in group settings, can also improve children's self-acceptance and acceptance of others. A qualitative systematic review of the mechanisms of change of school-based art therapies posits that the school context, the process of art-making and reflecting on the artwork were important components of positive change (35). Authors recommend art-based approaches for children who struggle to verbalize their challenges, difficulties, and emotions. However, to our knowledge, there is no evidence regarding the integration of art-based workshops to improve the emotional expression of children with SLCD. Noteworthy are some studies that explored music therapy to enhance motor skills of children with SLCD. While these approaches may be beneficial in promoting various components of language (36, 37), there is a lack of evidence on its potential benefits for this populations’ mental health.

Qualitative research has a unique potential for studying arts’ creative, embodied and artistic dimensions, with more emphasis on the individual's experience, while embracing complexity rather than reductionism (38, 39). In fact, there has been an increased call for the inclusion of participants’ voices in research on the use of creative arts in therapy (29, 38). Within the context of school-based art interventions specifically, the students’ and teachers’ perspectives on the art interventions have generally been excluded from the published research to date. Including these perspectives early on in the development of art-based interventions would arguably enhance the participants’ experience and researchers’ understanding of results (29).

### 1.3 Aim

The objectives of this qualitative study were to explore (1) the acceptability and feasibility of an art-based intervention for elementary school students with SLCD, (2) the perceived benefits of the intervention on children's mental health from the perspective of the students and the educational staff, as well as through observational data.

## 2 Materials and methods

### 2.1 Design

The initial stages of program development should rely on various forms of qualitative data to document if the intervention should be further refined to better reflect the needs of the population (40). As such, early research evaluating interventions does not need to include large samples both for feasibility and ethical reasons. Instead, it is recommended to include fewer participants and gain insight into their experiences of the intervention, as well as obtain their recommendations (40). Once an intervention is refined and initial testing done, its efficacy can be documented with larger samples and quantitative methods. However, at the time of study, the present intervention was still at its early development stages. The development of the program had been ongoing within the research team and previously piloted with smaller groups. As such, the present study allowed for the beginning of the next stage of documenting the acceptability and feasibility of the intervention with the population directly.

A descriptive qualitative design was used in this study. Data were obtained through individual semi-structured interviews with the students, the teachers and specialized education technicians, as well as through focus groups and observational data collected during the workshops. This design was implemented to examine the participants’ perceptions of the art-based intervention, acknowledging the subjective nature of their experience. This approach was guided by a pragmatic paradigm, focusing on what “works,” deriving context-dependent meaning through human experience (41). Our team chose a qualitative design based on the general recommendation that preliminary studies need to focus on the acceptability and feasibility of a new intervention rather than on its efficacy. This allows for changes to be done to the intervention's components before they are evaluated for their efficacy on specific mental health variables (42). This is not to say that the intervention's perceived effects (benefits/drawbacks) are not to be investigated, as the participants’ perception is central to evaluating its acceptability and feasibility. Hence, acceptability and feasibility studies aim first to explore whether an intervention can be done, how it should be done optimally, and if we should carry out further testing (43).

There is no consensus on the definition of acceptability within intervention research (44). The present paper follows the theoretical framework of acceptability (TFA) proposed by Sekhon et al. (45), whereby acceptability is “a multi-faceted construct that reflects the extent to which people delivering or receiving a healthcare intervention consider it to be appropriate, based on anticipated or experienced cognitive and emotional responses to the intervention” (p. 1). As such, acceptability integrates the attitudes, satisfaction, and perceived feasibility of a study by the participants who directly experience the intervention, stakeholders, and those who deliver the intervention (44). The TFA includes seven components of affective attitude towards the intervention: perceived burden, ethicality, intervention coherence, opportunity costs, perceived effectiveness, and self-efficacy (45) (p. 7). The feasibility of the intervention can be understood as its usability (46). In the present study, this included the perception that the intervention was doable, easy to implement/use, that the materials were appropriate, and that the time constraints were well adapted to the context (45). In short, feasibility answers questions regarding whether the intervention can be done and can be differentiated from a pilot study that evaluates aspects of a study design and procedures for implementing a future larger-scale trial (43).

### 2.2 Participants

Study participants included two groups: (1) children with SLCD and (2) school personnel. Twenty children with SLCD aged 8–12 years old from two classrooms participated in the activities, the focus groups, and individual interviews. Two teachers and two specialized education technicians participated in this study. To be placed in these classrooms, communication difficulties experienced by the children had to be identified as moderate to severe (based on the level of impairment and need of support) by the school psychologist and/or a speech pathologist. Most children from these classrooms also experienced comorbidity with either mild intellectual disability, ASD, ADHD, or oppositional defiant disorder; however, the list of comorbidities and severity was not disclosed to the researchers, and this information was shared verbally by the teachers, and thus we could not include detailed information on this. Special needs presented by the children included repeating many times in different words the instructions, wearing a remote microphone for students with hearing aids, and speaking clearly. Observations did not indicate severe speech impairments, as all of the children were able to share their thoughts and experiences.

The two classrooms were recruited from an ongoing partnership between the corresponding author and principal investigator of this study (CMH) and an elementary school in the province of Quebec, Canada. Both classrooms were adapted for the needs of children with SLCD (e.g., a full-time specialized education technician in class, smaller class size, etc.). The inclusion criteria for the school were to be a francophone or anglophone elementary school in the province of Quebec, Canada with specialized classes for children with SLCD. There were no additional exclusion criteria. Parents consented to their child participating in the art-based activities, focus groups, and individual interviews. Verbal assent was also obtained from the children for the focus groups and the interviews. Teachers and specialized education technicians who agreed to participate in the intervention conveyed their full informed consent to host the workshops and participate in a brief post-project interview. The present study obtained the approval of Bishop's University ethics board (102682).

### 2.3 Intervention

The arts-based intervention used in this project was developed by a child psychologist (CMH) and a Ph.D. student in clinical psychology (TLG), in collaboration with a researcher with extensive training in dance and movement therapy (AM). Themes were first chosen based on prior research projects led by the research team using art-based approaches to improve elementary school-aged children's mental health [see (21, 39)]. The choice of various art mediums was done in collaboration with both teachers taking part in the project. The 8-week intervention included different art mediums to address each weekly theme. Many complementary mediums were chosen to appeal to the different preferences of children, as well as promote various domains of creativity. As such, mediums included drawing with coloring pencils and crayons on white pieces of paper, as these mediums are easily accessible to children, and non-threatening. Crayons also allow for the exploration of perceptual components and symbolism within art (47). Play-Doh and Lego were chosen to encourage the kinesthetic components of art, which promote body awareness and release of tension (47). Further, these mediums are also familiar to children and easily accessible to allow a feeling of comfort and encourage creativity within cognitive development. Dance was also chosen to further bring consciousness to internal emotional experiences, promote emotional awareness and expression, as well as to release stress and tension (48). While music therapy could be beneficial for exploring emotions while promoting motor skills, it is recommended that these be led by a licensed music therapist with extensive training (36, 37). Further, with the limited time constraint, we wanted to repeat each art medium at least once to promote feelings of competence, so we limited the number of mediums included. The breakdown of the intervention mediums and themes can be found in Table 1. Drawing, sculpting (including the use of Lego and Play-Doh), and dance were selected to introduce a variety of accessible means of creation and expression for the students. Workshops were led by a clinical psychology Ph.D. student (TLG), who was assisted by an undergraduate psychology student, and both were supervised by a child clinical psychologist (CMH). Each weekly 50 min workshop around one theme was divided as follows: instructions, artistic creation with one medium, group discussion, sharing and presenting the creations to the group. The art-based activities were selected to encourage introspection, emotional awareness, and expression in children. The Canadian school curriculum for elementary schools already includes visual art classes. These classes aim to teach various art techniques and mediums and “To learn to create, interpret and appreciate artistic productions as a means of integrating an artistic dimension into their daily lives” (49). In this context, the children's visual art is evaluated for its content (coherence, organization, complexity of artistic elements). In contrast, the aim of art-based therapeutic approaches is to focus on the process of creation and less on the finished art piece (50). Children are rather encouraged to express themselves, focusing on various internal experiences (thoughts, emotions, physical sensations) related to art-making. As such, special care was taken during the intervention to differentiate these different creative spaces, emphasizing that the finished piece would not be evaluated, and children were never obliged to share them.

**Table 1:** Breakdown of the art-based activities.

Intervention week	Art medium	Theme
1	Drawing	Ugly drawing
2	Dance	Emotions
3	Sculpture	Family
4	Lego	Self-portrait
5	Dance	If you were an animal
6	Sculpture	Safe space
7	Lego	Something scary
8	Drawing	Recap of the activities

### 2.4 Data collection

In line with a pragmatic approach, data were obtained from four sources to triangulate the findings: focus groups after each workshop, observations during each activity, individual semi-structured interviews with the children and individual semi-structured interviews with teachers and specialized education technicians at the end of the intervention.

After each activity, the weekly focus groups were led by the first author of this paper and included questions about what the children had created, if there was something they wished to share with the group, how they appreciated the activity, if the activity was challenging in any way, and how the activity related to their everyday life. These discussions were recorded and transcribed (mean duration of group discussions = 8.15 min).

An undergraduate student in psychology, who acted as a research assistant, took observational notes during each activity based on an observation grid (see Supplementary Table 1). Questions from this grid included: *What are the reactions to the activity? What roles (leadership, listening, oppositional, etc.) did the children take? What were the interactions during the workshop? What did the children do or say*? The first author, who led the group discussions, also added any additional observations after the workshops.

After the eight-week intervention, each student participated in a brief individual semi-structured interview. These were recorded and transcribed (mean duration of interviews = 11.92 min). The interview guide included questions about children's perception of the activities, and if there were things they had not understood or would have been done differently (see Supplementary Table 2). The first author and a research assistant led these interviews in separate rooms in the school. Special care was taken when developing the interview guide to use words that were easy to understand and questions that were also easily comprehended. Further, children were told that questions could be repeated, that they could take all the time they needed to answer and find their words. The last drawing made (summary of the activities from the child's perspective) also guided the interviews, as children could point to elements on their drawing to describe their answers. Interviewers also took care to summarize what they understood from the children’s answers and ask them if it reflects what they experienced.

Finally, the teachers and the specialized education technicians from each classroom participated in semi-structured interviews in pairs (teacher with the class specialized education technician; mean interview time = 43.83 min). These interviews were conducted online on the Microsoft Teams platform (version 1.5.00.31168), housed on a secured server. Semi-structured interviews aimed to obtain the teachers’ and technicians’ perceptions of the intervention to find out what they thought had worked best and could be improved, as well as the perceived benefits of participating in these activities (see Supplementary Table 3). All interviews were recorded and transcribed for analysis.

### 2.5 Analysis

Given the clear criteria related to the acceptability and feasibility TFA framework of Sekhon et al. (45), content analysis was used to analyze data. This deductive approach to qualitative coding allowed for the application of a theory to the data (51). The transcriptions were analyzed using MaxQDA Plus 2022 (Release 22.7.0). Codes were first defined. Two researchers (MB and JP) then immersed themselves in the data, by reading and re-reading the transcripts, getting a sense of it as a whole (preparation phase); open coding of the codes was then applied. Interviews were then independently coded by a third researcher (TLG). A flexible approach was also used to allow any additional codes to emerge inductively. Next, all codes were grouped into meaningful categories through extensive discussions between researchers.

Following the deductive analysis, an inductive thematic analysis with a description-focused coding strategy was used to closely represent the participants’ specific behaviors, experiences and perceptions (52) to answer research questions regarding mental health. The method proposed by Braun and Clarke (53) guided the analysis. After the transcription and familiarization phase, one researcher (TLG) generated the initial codes, guided by the research questions. These initial codes were discussed with and reviewed by a second research team member (CMH). Three independent coders (TLG, MB, JP) then analyzed the transcripts using the same coding tree. Next, the codes were grouped into overarching categories representing themes within the data to ensure that they closely represented the voices of participants. The validity, coherence and consistency of these categories were discussed with the research team. Finally, overarching themes were named and defined. Data from the observation of the activities and the reflective journal were combined and used to add to the emerging themes from the interviews and focus group data. Hence, results present these themes that are rooted in the transcripts and closely represent what participants experienced and shared within this study.

## 3 Results

Thematic analysis yielded four overarching themes: acceptability of the activities, feasibility and implementation strategies, positive perceptions during the intervention, and valuable components of the workshops. For conciseness, the next sections present the themes that are anchored within the data and all supporting illustrative quotes are presented in Tables 2–4.

**Table 2:** Illustrative quotes of the construct of acceptability within the children and teacher interviews.

TFA construct	Illustrative quotes
Affective attitudes	“Well, I found it really fun” (Student) “I was really comfortable for sure” (Student) “Well, I would like to redo it all again with you!” (Student) “At the time, the children were very, very calm, much more than usual when they were doing their workshop there, if I can call it that, whatever it was; Lego or drawing or all that, I found that this time of day was much quieter than usual.” (Teacher) “You know, when they saw that Friday they had the art-intervention, they were like ‘yay they are coming, it will be fun’.” (Teacher) “You know, for me it was just really super. […] I would 100% recommend the program!” (Specialized education specialist)
Burden	“Well, we talked about it, I really liked being more of an observer of my students. Which I can do like, never, because I'm always planning the activity, leading it, and then, support them while they do the activity. And then [with the art activities], given that I wasn't planning anything, it was even a surprise for me when… you know, I saw at the same time as the students what you were asking them to do. And I was really more in the position of an observer, and I really liked that. And you know, we know our students very well, but there are still little things… Sometimes I was like, ah ok!” (Teacher)
Intervention coherence	“It was that there were no expectations necessarily at the end… It was just to create freely.” (Teacher) “I have nothing negative to say honestly, your animation was great, it was very simple and instructions too. It had to be simple for our clientele of students, you know reformulation to their needs…” (Teacher) “The choice of activities was really adapted to them [the students], I think they all had their favorite moment. Some really liked the dance, others it was more the Lego, so everyone say their favorite moment.” (Teacher) “Well, it was really clear [what to do]” (Student) “I understood everything.” (Student) “Because I'm used to drawing uh… to drawing well, and it was really hard [to do an ugly drawing].” (Student) “It took them away from their school routine to do arts… you know, have something different.” (Teacher)
Ethicality	“You know, it's not… well, it's not in our mandate… Yes, we have to ensure their well-being, the students, but it's not in our mandate, you know, to check everything that's… their emotions in depth, all that. You know, the purpose of school is really to educate, to socialize, and then to help them learn, and in this way, you know, we learn more about our students, but at the same time, you know, it lets them express things they don't normally have the chance to do in class. I thought it was really good to put that into… I think it's great to do that in a school” (Teacher) “Well, you know, I was already comfortable dancing because I used to do it. I'd been doing it since I was little and since I was older. Play-Doh, well also when I was little, well I practised, I like it, it, it… well, I like it” (Student)
Self efficacy	“It was easier when I had an idea [of what to create] right away…” (Student) “You know sometimes if we show an example at the beginning, well they will tend to reproduce it without really thinking about it. So, I found that good because you didn't show, you know your creation first, you know, *here’s my family,* because I think the results would have been biased a little or they would have wanted to reproduce what you would have done, so that's a plus to just give the instructions but without setting an example.” (Teacher) “Well, you know, at the beginning, in the activities, for you to have an example that you had done… but it was also ok because at the same time, it proved to the students that it doesn't matter, I can do it like that, and I like it like that. But it's okay to go with your creativity because you know, if I remember correctly, for the self portraits, you know, they were looking for all the colors, really… But, you know, if you had made an example of well, lots of colors, something funky, you know and tell them: “me in my body, I feel, full of colors, so I put lots of colors”, well it's… at least maybe they could have been like “Well ok, I can add yellow and orange and green… it’ll still be beautiful.” (Teacher)

**Table 3:** Illustrative quotes of the construct of feasibility within the children and teacher interviews.

Feasibility construct	Illustrative quotes
Time	“It took about one or two periods for the students to get to know you and feel comfortable. After they got to know you, they got into a kind of routine, and it was better then” (Teacher) “I would not have done less than one hour; we wouldn't have had time to really do the workshop.” (Teacher) “Your preparation time at the beginning was fast, you were really ready.” (Teacher) “I think that at the end we didn’t really have the time remaining to share the projects [I would suggest] 10–15 min of discussion. It leaves less time for creation, but at least 10 min.” (Teacher) “[It was a challenge for me to] have enough time to finish.” (Student)
Practicability	“I think that being in the classroom helped them to continue to flourish because when they change their environment, sometimes there is a blockage, but in class, they just felt that something else was happening in their environment, and they let themselves go there.” (Teacher) “Yeah, but I think that by doing it in their class, we really saw more of the true version of the students than if we had taken them out, brought them to another room, I think that there could have been students who would have been perhaps more reticent or in observation of what is happening around. Here [in the classroom], it was really like… in their environment, at their desk, with what they know around them, I think that helped them.” (Specialized education technician) “I found it [the intervention] very suitable for the environment of our class. You know, because they all had language disorders that were expressive but also receptive, the side of… your explanations and the use of the material, I found that really great.” (Teacher)
Material	“The material was on the table, and you know, you just had to give your instructions and then the students could begin… No, I have nothing negative to say”. (Teacher) “Maybe the modeling clay, the fact that they couldn't mix the colors… Maybe there's that, yeah… It complicated the project a bit because you know, they couldn't push on it to make it hold, so that it would be more solid, so sometimes it would fall. They had to find a way to make it fit without sticking it…” (Teacher)
Ease of implementation	“The students knew what to do, the equipment was provided, they were motivated…” (Teacher)

**Table 4:** Perceived mental health implications of the art-based intervention.

Mental health construct	Illustrative quotes
Emotional expression and regulation	“[art can be used for] expressing yourself a bit, like you know, some people express themselves through drawing, some through activities, some through dancing, stuff like that, but for me, to really express myself, you know when you're quiet, and you're sad, stuff like that, you can't talk about it with anyone, well, I draw all the time to take my mind off things… For me, it's… it's nice to draw and do activities with you. (Student) “[The art activities helped] To control my emotions a little, sometimes… I have a lot of anger… A lot of other stress and all that… I'm not able to control myself but it made me control my emotions more. […] I don't really know [how the activities helped that], but it was when we did an activity like Play-Doh, dancing, anything, well it I wanted to do it, but, if we didn't want to do it, we didn't… it was not obligatory, but really the activities were amazing!” (Student)
Feeling challenged	“The [ugly] drawing [was a challenge] because I always apply myself, but then, it made me feel good to draw a little bit whatever.” (Student) “I found it extraordinary to see this student dance when he is usually scared to raise his hand to speak up during class.” (Teacher) “We saw some students that it [dance] destabilized them, but they still participated, so it pushed them to go a little further than what they are perhaps capable of doing or that they were holding back on certain things… Butt then, it was letting go, given that it was in a context like a workshop with you, the two of you who were there…
Autonomy	“[art can be used for] expressing yourself a bit, like you know, some people express themselves through drawing, some through activities, some through dancing, stuff like that, but for me, to really express myself, you know when you're quiet, and you're sad, stuff like that, you can't talk about it with anyone, well, I draw all the time to take my mind off things… For me, it's… it's nice to draw and do activities with you.” (Student) “To just build something, well they really needed help from adults. So, it's certain that I imagine that the more we do it, the more you know, they are able to go back to certain memories or just build something with an idea in mind, reproduce it, come out a little to put it visually, because yes they think about lots of things, but to put it on paper, on Lego, on in movement… It's more… it's more complex, than they are used to, you know, working, with words, with numbers, but going like… yeah, finding ideas, memories, emotions… I think it makes them more autonomous after that, to perhaps come and express themselves, and recognize their needs or their feelings.” (Specialized education technician)
Competence	“In a way, it [creating art] increases their self-esteem, and we have several in class, you know, whose self-esteem is really, really low, but to do something like *Well, I managed to put into images what I was thinking, and it’s beautiful, I've succeeded*, well, listen, it makes them feel much more competent.” (Teacher) “Ok, well let's say I take [names a student], it's difficult for her to talk about her emotions, so do art therapy, and build herself out of Lego blocks or make her safe place, I find that it was easier for her because she used creativity, and she's super creative so that was really a plus.” (Teacher) “[I feel] More capable of making Lego” (Student) “Interviewer: Can you give me an example of an activity that made you feel competent? Student: The dance!”
Relatedness	“Well, it was fun, all the friends participated at the same time… You were there, you know, I felt at ease” (Student) “For me, it's pleasant, and you know, it's fun to do activities like that with other people, and things like that, because it's true that it's boring when people don't participate… So yeah, it’s fun to participate together.” (Student) “When did they made a semi-circle and had to stand up… oh to go do their choreography! Through the eyes of others, to see a smile, I think to really have everyone's eyes on their own person, well I think that that can actually help to feel connect… because yes, they are sitting in the class, yes, we experience moments, we do workshops, we have, we have lots of varied things in the class, but that's it, they were like under the spotlight, if I can say so. Then to be applauded, to be complimented: “Wow, what you did is beautiful, how did you think of that? » You know, that's it, it can just come, awaken lots of beautiful things in them.” (Teacher) “Yeah, you know, it's saying *Well you know, I made my family, but my family doesn't have many people because,* a bit like [names a student], *but I'm in a foster family, So, my family doesn't count in that.* Well, for her, it's difficult, but to say like, *I made my big sister because she's my only family*, well at the same time, she opens up to others to say: *me, this is my reality, and it makes me experience emotions*. To have this openness, it creates a connection, to be vulnerable around other people who are also vulnerable in fact.” (Teacher)

### 3.1 Theoretical framework of acceptability (TFA)

Six constructs of the TFA were coded through the interviews with the children and the teacher-specialized education technician pairs, as well as the focus groups and observation notes. Results are summarized in Table 2 with quotes supporting the acceptability of the art-based intervention from both perspectives. The construct of opportunity costs of participating in the intervention was not identified in the interviews nor the observational data and thus is not presented. Furthermore, the perceived effectiveness construct is integrated within the theme of the perceived mental health implications of the intervention (Section 4.3).

#### 3.1.1 Affective attitudes

This construct reflects how the children and teachers/specialized education technicians felt about the intervention. In general, children had positive attitudes towards the art activities. They enjoyed the various mediums proposed and looked forward to the weekly activities. Students mentioned having fun and feeling happy during the intervention. Observational data also highlighted how the children seemed involved and comfortable as they were making art or sharing their experiences. Two students discussed that having the activities in their group with peers they already knew made them feel particularly comfortable from the beginning. Teachers mentioned that the class was remarkably calm and absorbed in the creative process. This state of ease in the classroom made teachers embrace the creative arts experience. Both teachers and specialized education technicians felt particularly grateful that the art created space for them to learn about their students. Many children and teachers spontaneously mentioned that they would redo the activities because they added value to their weekly routine.

In general, students enjoyed all the activities. When asked what their favorite activity was, no one activity nor medium emerged as a clear favorite. Many students mentioned liking all the activities and having difficulty choosing just one. Similar patterns emerged for the least enjoyed activities, with a few children identifying the dance activities as a challenge. This was not surprising, as the observational notes confirmed that it took over 10 min for the students to start feeling at ease during the dance workshops. However, the children mentioned that they overcame this challenge and felt proud of themselves for letting go of potential embarrassment. Observational notes also highlighted how, after this 10-minute mark, students seemed to let go and ease into the activity. Noteworthy, dance workshops were particularly appreciated by the teachers and specialized education technicians who said that they could observe their students “come out of their shells.” The most reported words describing the general experience of the art-based activities were *fun*, *cool*, *enjoyable*, and *loved it*. No neutral or negative affective attitudes towards the intervention emerged in the interviews.

#### 3.1.2 Burden

The construct of burden refers to the perceived amount of effort required of the participants to participate in the intervention. Burden was infrequently identified in the interviews. Instead, the teachers and specialized education technicians all mentioned how having a period of art creation allowed them to take a step back and observe their students from a different perspective.

One teacher mentioned that they often feel overwhelmed by the curriculum and thus chose to concentrate on academic and didactic priorities. However, they also reported feeling responsible for attending to their students’ psychological needs and promoting their psychological well-being but did not feel they had the time or resources to do so. For them, observing a novel and accessible way to discuss emotions through art, removed some burden associated to caring for the psychological well-being of their students. As per the quote in Table 2, one teacher even mentioned having started to use drawing techniques when a student was having a hard time expressing themselves. Overall, the intervention was not perceived as giving rise to a burden; rather, it facilitated verbal expression and gave the teachers tools to explore emotions with their students. Importantly, there was no harm observed by the facilitators nor reported by the students, teachers, and specialized education technicians.

#### 3.1.3 Intervention coherence

The construct of intervention coherence refers to the extent to which the participants understood the intervention and how it worked. Observational notes indicate that the children had considerable experience with art-making. Many mentioned taking dance classes, making Lego at home, and enjoying drawing in class. The particularity of art-making in the context of this intervention was that it did not aim for a finished piece but rather to embrace the process of creation. This concept was rather well understood by the students, while some mentioned feeling frustrated at times being unable to finish their creations. However, participants were generally able to let go of their expectations. Further, when asked if the instructions were clear, all participants mentioned that what was expected from them was always well explained and explicit. This also stood out in the observational data, as children always went right into the creative process after the instructions were provided and created meaningful pieces using the art medium provided.

Teachers understood the intervention well, mentioning some key concepts during the interviews, such as the importance of creating a space without expectations, in order to create freely. Further, both teachers and specialized education technicians said the instructions were very clear. They appreciated that these were simple for their students, who generally found instructions complicated and difficult to understand due to their language difficulties. Instructions were at times repeated or rephrased to allow for better understanding, which teachers also acknowledged as important and well-conducted. Furthermore, teachers also appreciated the opportunity to break their students’ routines and offer workshops that did not aim to enhance academic learning directly.

#### 3.1.4 Ethicality

The construct of ethicality in the TFA framework refers to the extent to which the intervention fits well with an individual’s value system. The present art-based intervention seemed well suited to the teachers’ and specialized education technicians’ values to integrate emotional expression and awareness in the school. Teachers and specialized education specialists were particularly grateful that a safe space was created, and that sufficient time was put aside to do this, as they reported often feeling rushed in their daily routines to do so. Thus, the art intervention put into action the teachers’ value to integrate emotions in the classroom. Further, art-making was observed to be rooted in the children’s interests. They mentioned many times that they had already made Lego, sculptures, drawings, and dances, indicating that the intervention included activities that were accessible. Overall, the intervention seemed to be suited to the school, the teachers’, and the students’ values, keeping them interested and making the emotion-based art-intervention meaningful to them.

#### 3.1.5 Self-efficacy

The construct of self-efficacy refers to the participant’s confidence that they can perform the behavior required to participate in the intervention. Observational data indicates that participants not only engaged in the art-based activities, but that they were also able to create within the suggested themes. Some participants initially struggled with finding ideas, taking their time to choose what they wanted to represent with their creation. These students requested support from the facilitators or their teacher to talk through ideas and validate the ones they might choose to create. One teacher suggested that in order to foster self-efficacy, examples could be provided to show the potential of creation within each medium and each theme. However, the other teacher did not agree, as the students tended to copy the example instead of applying the theme to their experience. Factors that seemed to facilitate self-efficacy included having age-appropriate instructions, using art mediums children were already comfortable with, and giving individual support when needed.

In summary, the perceived acceptability of the intervention relied on positive affective feedback throughout the intervention, no perceived additional burden, a good understanding of the intervention, a good fit within the school context and feelings of self-efficacy.

### 3.2 Feasibility framework

Content analysis of the interviews, focus groups and observational data revealed the four categories from the TFA. Each is presented below and in Table 3 with the supporting quotes from the interviews.

#### 3.2.1 Time

The concept of time can be important in the school context because the schedule needs to be respected: the intervention had to be conducted in the right amount of time, which was a 50 min period. This relates to allowing enough time for art-making as well as keeping all children interested and active. Teachers agreed that a one-hour period was the right amount of time to conduct each workshop (introduction, instructions, art-making, discussion, clean up). However, one teacher suggested that more time should be allocated to the discussion period and sharing the art. Indeed, the discussions lasted between 4.70 and 11.58 min (mean discussion time = 7.26 min), and observational data confirms that these were sometimes shortened owing to a lack of time. The teacher mentioned that the art-making could be done in 40 min, allowing a 10–15 min discussion. Observational data suggests that most students would finish their pieces in 45 min. One student mentioned in the interview that they did not feel like they had enough time to complete the Lego sculptures.

Relevant insights from teachers include the importance of allowing a week or two for the class to feel comfortable with the first author and the research assistant leading the workshops. This was also observed during the session, whereby the students felt more comfortable sharing their art by the third session. This was particularly obvious when comparing the first and second dance workshops. The children were shy during the first dance workshop (week 2), especially for the free dance and improvisation components. They would do minimal movements and look at others and the adults for approval. They were much more comfortable by the second dance workshop (week 5), allowing bigger movements and exhibiting increased confidence. Hence, a recommendation might be to consider moving the first dance workshop later in the intervention to allow this confidence to be built.

#### 3.2.2 Practicability

The concept of practicability refers to elements indicating whether the intervention was doable from the perception of the students, teachers and the people leading the intervention. Observational data suggested that the intervention plan was well followed and was not changed at any time. The activities were all conducted in the intended way and did not need to be further adapted. Both observational data and teachers/specialized education technicians interviews indicate that the intervention was easy to deliver, as the activities were well explained and well understood. Teachers also mentioned that using the classroom to complete the activities added to the intervention's practicability. As the quotes in Table 3 highlight, teachers and specialized education technicians believed that students were more comfortable in their classroom environment, making the implementation easier. Furthermore, the practicability of the intervention was also established by having researchers lead the activities. Teachers mentioned that having nothing to prepare was particularly appreciated, as their schedules were already full. In summary, the intervention implemented in the classrooms and facilitated by the first author and a research assistant was doable.

Furthermore, regarding practicability of the intervention, teachers recommended that parents be further informed and included in future iterations of the project. For example, one teacher suggested that the children's art be shared with the parents either by email or with a printed picture. They highlighted how this could create a space to further discuss these themes at home and practice communication through art with parents.

#### 3.2.3 Materials

Materials were a core component of this art-based intervention. The researchers brought the necessary materials for the scheduled activity each week. These included Play-Doh, Lego, pipe cleaners, scarves, soft balls, and crayons. The materials were set up at the teacher's desk for the class to come pick up during the activity. Students could hence pick their colors, shapes, or other attributes of the materials provided to them. Observational data suggested that students were excited to be able to choose their colors, and this added to their feelings of creative liberty. Teachers also mentioned that the materials were suitable for the class. One of the challenges observed was that the different colors of Play-Doh could not be mixed, making the sculpting difficult at times. This decision was made so that colors could be reused in the other classes and the different activities. Nonetheless, one teacher suggested that one color be given to each student and that they make their sculpture with that color only. This challenge did not emerge from the interviews with the children, and the observational data suggests that sculpture solidity issues were overcome with the use of other objects, such as cardboard. In summary, the materials used in the intervention seemed to be appropriate, easy to use, require little setup and cleanup costs, of good quantity and well organized.

#### 3.2.4 Ease of implementation

Ease of implementation relates to how the intervention was applied in the school context. This concept was closely related to the practicability of the intervention, as teachers greatly discussed the implementation in their school context. As previously mentioned, teachers highlighted the fact that outside researchers came into the class to facilitate the art activities made the implementation effortless for them. They repeated that they appreciated not having to prepare the activities and having the space to observe their students in a different context. Further, observational data highlights that the overall implementation went smoothly. The classes were ready to welcome the researchers at each of the reserved periods, the students were prepared for the activities when the facilitators arrived, the participants understood the instructions, and the art-making was executed within the desired parameters (i.e., although there were no expected outcomes, the children all made a meaningful piece of art that represented the theme of the week in their own way). The chosen themes were relevant to the students, as they elicited positive reactions, were a stimulus for mental health conversations, and enabled the creative process. Only one student was not able to let themselves make something scary, even if discussed with the facilitator and the teacher. This student's teacher mentioned in the interview that this was coherent with this student's attitude in class and their limits. In this case, the facilitator suggested the student did something that could be scary for someone else, and the student was able to let themselves create something within these parameters. Overall, the intervention was well implemented in the school context, given the facilitators’ preparedness and past experience leading activities with elementary school children and the teachers preparing the classroom.

In summary, the interviews and observational data suggest that this intervention was feasible in the school context as the time constraints were respected, the intervention was doable, the facilitators brought the materials, and the context allowed a smooth implementation.

### 3.3 Mental health implications

Thematic analysis yielded the overarching theme of the mental health implications of the intervention. The sub-theme of creative exploration emerged as being related to these perceived benefits. Creative exploration was defined as using art and creativity to explore emotions, thoughts, themes, and ideas about the world. This led to perceived changes in children in the domains of the self and with others in the classroom setting. Both these sub-themes are discussed below and supporting quotes can be found in Table 4.

#### 3.3.1 Creative expression and the self

The first sub-theme that emerged from the data was the theme of creative expression and the self. Notably, the categories of perceived changes included enhanced emotional expression, as well as changes in feelings of competence and autonomy.

##### 3.3.1.1 Emotional expression

Given their SLCD, verbal emotional expression was difficult for most of the children. The teachers noted that having another medium to express their emotions helped many children open up. Notably, one extremely shy participant was able to discuss their fear of monsters by sharing their artwork during the focus group. As discussed above, the teachers mentioned that they learned a lot about their students throughout the intervention. For instance, they learned about their students’ families and fears, as children were able to articulate these through the creative process. Teachers and specialized education technicians mentioned that they would have never known about these experiences or emotions otherwise. Student responses from the semi-structured interviews revealed that many felt an increased ability to express themselves in class by voicing their ideas and opinions. This also related to the feelings of autonomy, as participants felt an increased sense of space for their ideas and emotions, as supported by the quotes in Table 4.

Observational data also suggests that children expressed feelings through their art in relation to the weekly theme. Further, in the focus groups, students could articulate their feelings verbally, mentioning how they felt during the art-making, and identifying how they felt related emotions in their body or what caused these emotions to emerge.

##### 3.3.1.2 Feeling challenged

Teachers reported the children's ability to go out of their comfort zone, mainly during the dance workshops. One of the teachers mentioned that they were impressed by how the children took a risk and danced in front of their classmates. They also noted the challenge students faced when expressing a particular feeling with a movement. One of them mentioned that: “even for me, I was like, oh boy, how am I going to express my emotion in a movement?” The teachers also mentioned that some of the children seemed destabilized by the dancing activity, but still participated, which took courage on their part. One teacher expressed how impressed and amazed they were by one student during the dancing activity: “I found it extraordinary to see this student dance when they are usually scared to raise their hand to speak up during class.”

Although some of the activities (e.g., dancing emotions and creating something scary out of Lego) presented challenges to several students, they were able to overcome these. For example, when one student was asked whether they experienced any difficulties during the activities, their response conveyed a desire to learn from these. Moreover, two students cited the “ugly drawing” activity as the most challenging given that they had become accustomed to drawing aesthetically and caring about the visual outcomes of their artistic endeavors. One of these students reported that this activity was challenging; however, the second observed that “it made [them] feel good to draw a little bit of anything.” Hence, this student was able to experience a pleasurable feeling that arose from their willingness to venture out of their comfort zone by wholly engaging in the “ugly drawing” activity.

##### 3.3.1.3 Autonomy

The concept of autonomy emerged from the interview with the teachers. One of the teachers mentioned that being autonomous was an issue for several students. Many of them relied on their teachers to find ideas of what to create, as well as express their thoughts and emotions. Teachers’ discourses indicated that children had little space to experience a sense of volition in the classroom. Hence, at the beginning of the intervention, some students required help to identify their values, interests and beliefs that may drive their choices. As the intervention advanced, they embraced this space of choice in creating their artwork.

Observational data suggests that some children felt the liberty to be themselves and to create volitionally according to their values to be overwhelming, as they asked many questions and took a long time to start their creation. For others, autonomous decision-making was welcomed, and they would jump right into the activity. As the intervention progressed, it was observed that fewer students would ask for support in the decisions related to what to create. The concept of autonomy was observed when children chose what art they wanted to create, the colors they preferred, and how they would represent what they had in mind. Students’ interviews reference increased feelings of freedom in being their authentic selves, as they felt the liberty to express what they wanted and how they wanted to express it within the parameters suggested by the workshop activity.

Furthermore, the art-making process allowed some participants to express and externalize their emotions. For instance, one student found that engaging in arts and crafts enabled them to express themselves and their ideas better. Moreover, another student mentioned how feelings of joy surfaced following the weekly art activities. They subsequently mentioned that they felt free to be themselves in the context of these activities and their classroom. Children discussed emotional expression concerning feeling free to be themselves in the presence of others. Further, observational data suggests that the artistic process enabled children to work through differences and insecurities, facilitating their emotional expression in the presence of others and ultimately allowing them to be the most authentic version of themselves. The teachers noted that the children could use their creativity to make art that represented how they felt, which facilitated working through difficult emotions or themes.

##### 3.3.1.4 Competence

The concept of competence was tightly linked with a sense of accomplishment and self-confidence. As opposed to the usual academic activities (e.g., languages, math), teachers and specialized education technicians mentioned that the art-making process did not require a right or wrong answer, leading the children to use their creativity to initiate work without any performance-related considerations. Data from teachers and observations also show that children were proud of themselves and wanted to share their creations with their classmates and teachers. As one teacher mentions:

In a way, it [creating art] increases their self-esteem, and we have several in class, you know, whose self-esteem is really, really low, but to do something like “Well, I managed to put into images what I was thinking, and it’s beautiful, I've succeeded,” well, listen, it makes them feel much more competent.

Most student responses about competence related to improved abilities in specific art activities. For instance, one student explained how, by practicing and observing other students, they could improve their drawing skills. Similarly, another student stated they felt more competent in art activities involving drawing, Lego, and play dough. Overall, the students felt they could gain a sense of mastery, especially as there were two different sessions involving the same medium. As for dance, observational data showed that the children were rather shy and closed off at the beginning of the first workshop exploring this medium of expression. However, by the end of this first period and throughout the second dance workshop, students became more at ease and let themselves explore various movements. This was related to feelings of pride; for some, this medium of expression was both the most challenging and the most appreciated. Overall, children's discourse, teacher/specialized education technicians and observational data indicate that art-making was related to perceived feelings of competence in some students.

#### 3.3.2 Creative expression with others

The second sub-theme that emerged with regards to mental health was creative expression with others. This referred to the experience of relatedness brought by the creative process and the discussions.

##### 3.3.2.1 Relatedness

Students’ feelings of relatedness with others appeared to be related to their motivation to participate in the art-based activities and were indicative of their level of comfort during the activities. This became apparent when one student partially attributed his incentive to engage in these activities to the fact that he had “fun with his friends” while doing so. Similarly, another student ascribed their pleasurable experience of the creative workshops to the act of “participating all together” and enjoying joining in activities with the full classroom (including, but not restricted to, their friends). Other students specifically expressed that knowing those who surrounded them during the art-based intervention enabled them to feel at ease. In sum, the data suggests that creating art in a group increased the students’ perceived feelings of comfort, potentially inciting greater emotional expression.

Furthermore, one teacher mentioned that students opened up during the group discussion and seemed more welcoming of the experiences of others. They discussed how the intervention made the children interact throughout the group discussions and were impressed by how open the children were with their classmates. This was also apparent through observational data that noted that children would react positively and supportively when others would share their art, reflecting on similarities and differences, as well as general appreciation.

Teachers also noted a fear of being judged that was an issue during some activities, notably the dancing activity. However, all students took the opportunity to experiment with dancing at their own pace. Throughout the intervention, the children gained confidence and let go of their fear of how others would perceive them. Teachers reported that it took courage to be under the spotlight and to share their experience and creations. It was also gratifying for children to get applauded and complimented for their art creations. Observational data further highlighted that student interactions were positive, accepting, and supportive. When asked how art activities could help in school settings, one student responded that it could assist students with less verbal fluency or second-language speakers in expressing themselves. This suggests that students taking part in the art activities were accepting of others and recognized the value of artistic interventions in facilitating the integration of all students in their classroom.

## 4 Discussion

The present study aimed to explore the acceptability, feasibility, as well as students’, teachers’, and specialized education technicians’ perceptions of the benefits of an 8-week school-based art intervention aimed at supporting the emotional well-being of elementary school children living with SLCD. The overarching themes were those of the perceived acceptability of the intervention within the TFA, the perceived feasibility, and the perceived mental health implications.

### 4.1 Acceptability and feasibility

Overall, the findings suggest that the school-based creative art intervention was acceptable and feasible for children experiencing SLCD. Notably, components of the interventions that reinforce previous findings regarding school-based interventions include using age-appropriate approaches (54), reiterating that the goal of art-making is not the final art piece but the process itself (55), allowing enough time for discussions through the art (56), and choosing a variety of accessible mediums to discuss emotions (57). The results also indicate that the intervention was not perceived as a burden in the classroom. To this point, it is essential that interventions integrated into the classroom avoid teacher burnout (58). Further, one of the main goals of an acceptability and feasibility study is to determine if further testing should be conducted (43). These findings indicate high levels of these two constructs. Paired with support for the intervention from the students, the teachers and specialized education technicians, this suggests that the present intervention should be further evaluated using experimental methods. Finally, the results also indicated that more time should be given to discuss the art creations after the workshop. This element is essential to the process of change during art-making (56) and should be better integrated into future studies using art-based approaches.

### 4.2 Self-expression and the creative process

Although this study did not quantitatively measure the impact of the art-based intervention on the well-being of children with SLCD, perceptions gathered from students themselves, their teachers and specialized education technicians, as well as from the observation grids, tend to support the fact that the intervention facilitated self-expression, fostering self-confidence and creativity. As discussed previously, students initially felt challenged to express their emotions. However, the intervention—and the safe space created in its implementation—encouraged them to share their emotional and internal worlds through their artwork. This approach enabled them to feel more capable of expressing themselves, particularly because there was no concept of a “right” or “wrong” way to participate, which helped develop their perceived confidence, competence, and pride in themselves. These findings align with previous research highlighting the benefits of art-based interventions for individuals who have difficulty expressing themselves verbally (21, 22). Indeed, a study with preschool children with communication difficulties reported similar results, where movement, visual art-making, and theatre provided a space to communicate emotions in these younger children (12). These art-based activities in turn, reduced anxiety in children and increased their empathy (12). It thus seems that art-making could foster the emotional expression of children with SLCD within the classroom. To this end, parents of children with SLCD have expressed the need for mental health interventions that do not rely on verbal communication (59). It appears that art-based interventions could potentially serve this purpose.

While some students initially expressed frustration at letting go of perfectionist ideals, this challenge represented an opportunity to step out of their comfort zones and relinquish the notion of an aesthetic final piece of art. Since the focus of the activity was more on the creative process than the final creation, children with SLCD were freed from these expectations. They could concentrate on self-expression and enjoying the activity. This ultimately made them more comfortable expressing themselves and gaining confidence in their artistic abilities, as emphasized by various other studies (21, 22). Introspection and self-expression are fundamental to the therapeutic process of art-making, as this is believed to be a mechanism of change by which art-making can support mental health (60). However, this process of introspection and self-expression through art might not be accessible to children in all contexts. An approach that may be beneficial when young people are hesitant to open up to the creative process is philosophy for children (P4C), which could be useful to encourage young people's questioning about the work they are asked to create, thus enriching the creative process itself (61). Within the creation of art, combinations with P4C would make it possible to explore reluctance linked to performance anxiety (e.g., discussing what beauty is) and the fear of the gaze or judgment of others (61). It could therefore offer children a space to explore their doubts related to the creative process. In doing so, adding a P4C component would act as a catalyst to carry out artistic activities. Our own research suggests that combining P4C and other approaches may increase its benefits, as it helps children create meaning from their experiences while supporting their self-determination (62).

### 4.3 Basic psychological needs satisfaction

One of the most salient results from this study lies within perceived changes in satisfaction of the basic psychological need for autonomy in students, which can be linked to increased self-expression. Indeed, children reported that the freedom to express themselves authentically through their artwork, along with the absence of pressure to produce something aesthetically pleasing, contributed to building a stronger sense of self and to their feelings of acting volitionally, in accordance with their true self and thus in a more self-determined manner. While the need for autonomy is often referred to as an internalized and relatively hard-to-observe need (63), the fact that teachers and facilitators observed these changes could indicate that this was an essential aspect of the intervention. Interestingly, this diverges from other studies suggesting that teachers may not be the best proxy to identify changes in their students’ autonomy (64). Future research should investigate in what situations teachers or parents could provide valid evaluations or observations of changes in the satisfaction of autonomy in children. Furthermore, offering a variety of materials with which to create and sufficient time to do so allowed students to select what resonated with their thoughts and their envisioned creation, thus encouraging their autonomy.

Although a distinct concept from autonomy, self-confidence can contribute to feeling more autonomous and competent (65). Noteworthy, previous research evaluating the impacts of art-based interventions on mental health has also shown that artistic creation can enhance self-confidence in children (66). Other initiatives, such as the YouCreate program, have highlighted the essential role of the creative process in the journey towards self-discovery, self-expression, and acting in coherence with one's values (67). Further, children with emotional and behavioral difficulties who participated in school-based dance and movement psychotherapy reported that it encouraged self-expression, emotional regulation, mastery and acceptance of emotions, improved self-confidence and self-esteem, reduced stress, and helped develop positive relationships; all of which are essential in feeling self-determined, competent and affiliated (68).

Moreover, art-making promoted a sense of trust between students, their peers, and their teachers, thus contributing actively to the satisfaction of their need for relatedness. This echoes other research that found that an art-based intervention could increase empathy and connection between preschool students (12). Indeed, fostering a safe space for students to share their emotions with their teachers can positively impact their relationships. Many studies highlight the benefits of positive student/teacher relationships in students’ academic, behavioral, and social-emotional spheres (69–71) and consequently on the satisfaction of their basic psychological needs (72). Moreover, because the art intervention occurred within a group setting, it facilitated active participation and the development of social skills. Some participants mentioned how the activities also mitigated the language barrier challenge across students, facilitating the affiliation process. As a result, children felt more at ease expressing their emotions and being themselves in the company of others. These outcomes align with previous work based on self-determination theory, which emphasizes the role of relatedness in promoting psychological well-being (63). In this study, the sense of community and trust established through the activities nurtured a sense of security, authenticity, and belonging among the students, their teachers and specialized education technicians. This also resonates with literature on the importance of autonomy support in the classroom, which indicates that warm, supportive relationships could reduce students’ anxiety and depression levels (73, 74), while also promoting better adjustment (75). As such, feeling connected to other students and their teachers through the art-making process, while feeling supported in their autonomy in doing so, could be mechanisms of change in art-based interventions (76).

Finally, students felt that art-making and emotional expression led to increased feelings of competence. Indeed, it has been shown that engaging in accessible, yet challenging activities can contribute to feelings of competence in children, as they can observe their skills improve. With appropriate support, children can learn skills that are just out of their comfort zone, a concept that has been named scaffolding in the zone of proximal development (77). Introducing activities that are “optimally challenging” allows students to test and broaden their abilities (78). Art-making specifically can be a way of offering this means of support to scaffold children's introspection and emotional expression (79). In the context of this study, children who typically had a hard time expressing their emotions were provided with a new way to articulate their feelings with the support of both the mediums used and the facilitators. Hence, art-making perhaps contributed to enhanced feelings of competence, not only in creating art but also in the overall emotional expression. Art-based activities are flexible approaches that can be well-suited to meet children at their unique developmental stage through the variety of mediums and themes (80) allowing for feelings of competence to emerge.

Overall, feedback from students, teachers, and specialized education technicians alike all highlighted how the intervention allowed the creation of a safe space for individual introspection and emotional expression in the classroom. This, in turn, facilitated creativity and authenticity in children. Further, concepts pertaining to familiarity and comfort were considered significant factors supporting the acceptability and feasibility of the intervention, as indicated in the TFA's intervention coherence and ethicality components. For instance, students mentioned that they had already engaged in similar activities at home, which made them feel more at ease (thus more familiar) when participating and expressing themselves emotionally in school. These findings align with previous research highlighting the need for educators to include non-academic elements, like emotional expression, within the curriculum (81). Taking part in the intervention reinforced teachers’ conviction that it is important to consider their students’ emotional well-being in their teaching while providing a resource to do so. Children have been found to thrive in environments where their social-emotional skills are nurtured, and they feel self-assured, relaxed, and secure (82). Thus, it appears that results from this study support the premise that art-based interventions could support the satisfaction of the three basic psychological needs for autonomy, competence, and affiliation, contributing to overall mental well-being in children.

### 4.4 Implications for teachers’ well-being

Both students and teachers provided favorable feedback in terms of ease of implementation, time efficiency, and practicality. Teachers also expressed appreciation for the help of an external host overseeing the activities, allowing them to actively participate and engage in the process. They even reported indirect perceived benefits of participating in the activities on their own mental health. Although we did not plan the intervention with any benefits for teacher mental health, these results may hold promise. Indeed, it is well known that teachers often face multiple layers of stress that make them more vulnerable to burnout, mental, and physical health issues, as well as strain their relationships with students (83). Results from this study lead us to believe that developing art-based interventions, whether in individual or group settings, could be a valuable resource for teachers themselves and have trickle-down effects on their students. However, further research on this topic specifically is warranted.

### 4.5 Recommendations

One recommendation of interest raised by teachers was to enable the sharing of the created art with the children's families at home, to facilitate parent-child communication about school activities. Indeed, the research linking parental involvement and communication in their child's activities has established a clear causality with children's development, academic success, and emotional well-being (84, 85). Although many studies have explored the benefits of family-based art therapy, there exist no indications to our knowledge on the question of sending pictures of the students’ creations to their parents/guardians. Ethical considerations raised by the research team on this topic involve obtaining children's consent before sending these pictures and the limited emotional support that could be extended at home. Indeed, the variety of family dynamics and realities may bring about unexpected challenges. Nonetheless, the benefits of using the created pieces to continue discussions, emotional expression at home, and their inherent ethical and practical issues, should be further explored. In the meantime, organizing an exhibition of the students’ artwork after the intervention could serve to present creations to families and the community, drawing inspiration from photovoice research (86).

### 4.6 Strengths and limitations

This study counts notable strengths. It presents and integrates the rich perspectives of children and teachers on the effects of an art-based intervention on acceptability, feasibility, and mental health, through semi-structured interviews, focus groups and observational data. The qualitative design selected for this study allowed participants to voice their reflections on the process and the perceived benefits of the intervention and directly share their experience in their own words. Children's perspectives are often not directly taken into account in published research, while they provide valuable information about their lived experience (29). This is especially true in the context of art making. As such, a considerable strength of this study resides in giving them a voice about the perceived benefits of the intervention, while triangulating their experiences with other sources of data. However, like all studies that rely on participant self-reports, potential desirability biases may have been at play. Observational data and other strategies were used to reduce this bias (87), but it is possible that the children and teachers were biased in reporting their perceptions about the intervention. This may also have been brought about by the dual roles of facilitators and researchers, which could have hindered the expression of negative perceptions of the intervention. This was partly overcome by presenting an open attitude with a genuine desire to improve the intervention to benefit other classes and children.

Another limitation lies in the identification of the children with SLCD included in this study. Indeed, the diagnosis was given by the school psychologist, who would determine if the child was eligible to be in the special needs classroom. However, the researchers did not have access to these diagnoses and did not know the severity of the SLCD of the children nor the exact prevalence of comorbidities. Hence, the transferability of the results may also be compromised by this limited information about the participants. Furthermore, in the researcher's observation, the children had a relatively high level of verbal expression, and communicated with a wide range of vocabulary, as can be seen in the chosen quotes. These were representative of the language level of the participants. Most challenges resided in receptive functions and only required speaking loudly, articulating clearly, and repeating at times. Nonetheless, obtaining verbal information from the population of children with SLCD remained a challenge for this study and constitutes a limitation, as children were perhaps not able to express everything that they desired during the interviews. Body language and general reactions were noted in the observational data to allow for further understanding of the children's experience beyond what they expressed verbally. Several children asked questions during the interviews, asked for certain words to be defined or repeated, and expressed themselves when they did not understand questions. These problems were addressed by using different wording, allowing the children a certain amount of time to express their thoughts, and using reflection to ensure comprehension. Upon reflection, diverse strategies that go beyond verbal expression in the interviews could be used, such as drawing and miming techniques (88). Member-checking involves checking with the participants whether the results reflect their experience and seem valid or accurate. This approach could also be used with children in future studies to ensure accuracy of results, and whether these resonate with the participants’ experiences to ensure the credibility of the results (89).

Furthermore, it is strongly recommended to continue evaluating factors associated with acceptability and feasibility over time, as some longer-lasting perceptions may be relevant (90). Implementing a longitudinal design would be recommended. Finally, the transferability or social validity of the present results remains limited to the specific population (children with SLCD) in a relatively small sample within a particular setting (elementary school in Quebec, Canada). The present school-based research had limited access to some information that could have given a better portrait of the samples (e.g., socioeconomic data). It is recommended to find ways to access and present this data without compromising the confidentiality of participants to gain a better portrait of participants. Future experimental studies, employing both qualitative and quantitative designs, are needed to better understand the extent of the impacts of art-based interventions on student mental health in elementary schools, specifically those with SLCD or in special education classrooms across various age groups, socioeconomic groups and for children with various comorbidities. Positive findings in the present work indicate that the intervention effectively addressed the needs of participants. However, the same intervention themes and mediums may not be relevant to all youth populations. Indeed, the adaptability of art therapy enables researchers to cater their approach to meet the unique needs of participants (28, 91). To ensure an intervention is culturally appropriate, it is strongly recommended to include community partners in the research process, particularly in the creation of an intervention (92).

## 5 Conclusion

The present study explored the acceptability, feasibility, and perceived mental health benefits of a school-based art intervention in specialized education classrooms for elementary school students with SLCD. Results from this study show a promising potential of art-based interventions to enhance the mental health and emotional well-being of elementary school students living with SLCD. The findings, derived from in-depth qualitative assessments encompassing the perspectives of students, teachers, and specialized education technicians, underscore the feasibility and acceptability of such interventions. All data sources converged on the tangible perceived benefits, including improved emotional expression abilities, along with heightened feelings of autonomy, relatedness, and competence.

These outcomes reaffirm the transformative power of creative arts in fostering positive self-concepts and facilitating constructive emotional outlets. The art-based workshops were perceived as safe spaces where students could share their emotions and gain a sense of accomplishment through the creative process. Additionally, teachers’ newfound insights into their students’ emotional experiences highlight the potential of art-based approaches to cultivate more empathetic and constructive classroom environments.

However, while this study provides compelling qualitative evidence, it is essential to recognize the need for further rigorous experimental studies to corroborate these findings and establish a robust causal link between art-based interventions and improved mental health outcomes. Nevertheless, the present research lays a strong foundation for future endeavors aimed at harnessing the therapeutic potential of art to empower and uplift children facing communication challenges in educational settings.

## Data Availability

The datasets presented in this article are not publicly available due to privacy or ethical restrictions regarding qualitative data. Requests to access the datasets should be directed to Catherine Malboeuf-Hurtubise; catherine. malboeuf-hurtubise@ubishops.ca.
