# Supplementary material for: Feasibility, acceptability, and perceived benefits of a creative arts intervention for elementary school children living with speech, language and communication disorders
Source: Front Child Adolesc Psychiatry. 2024 Jun 5;3:1322860. doi: 10.3389/frcha.2024.1322860 (PMC11748800; doi:10.3389/frcha.2024.1322860)
Supplement: Supplementary file 1 [file Table1.docx]

| Observation guide | | | |
| --- | --- | --- | --- |
| Date: | Activity: | Start time: | Stop time: |
| **Area of observation** | **Observations** | | |
| What are the students’ reactions to the activity? |  | | |
| What are the students’ attitudes towards the activity? |  | | |
| What are the events that happened during the activity or notable events that influenced?  (E.g., interruptions, celebrations, etc.) |  | | |
| What are the different roles that children adopt during the activity? |  | | |
| What are the interactions between people? (E.g., nature of interactions, reason they occur, emotions expressed) |  | | |
| What are the relationships between the students during the activity? |  | | |
| What are people saying and doing? |  | | |
| What are the non-verbal messages? |  | | |
| What is the general atmosphere in each group? |  | | |
| How is the environment structured? (E.g., physical setting, sitting arrangement) |  | | |
