# Supplementary material for: Feasibility, acceptability, and perceived benefits of a creative arts intervention for elementary school children living with speech, language and communication disorders
Source: Front Child Adolesc Psychiatry. 2024 Jun 5;3:1322860. doi: 10.3389/frcha.2024.1322860 (PMC11748800; doi:10.3389/frcha.2024.1322860)
Supplement: Supplementary file 2 [file Table2.docx]

**Interviews with children – Semi-structures interview guide**

***Translated from French***

1. Introduction

- Description of the research project
  - As you know, we are doing a project to explore the effect of art activities in primary school classes. We wonder how the children liked it, if there are things to improve, how it all went for them.
- Description of the process and their participation
  - Some clarifications:
    - You don't have to answer questions that don't appeal to you
    - You can take time to think before you answer
    - Tell me if you don't understand a question
    - You can use whatever words you want to represent what you think and not what you think I want to hear; there is no wrong answer, what is important to me is to have your perspective
    - Respect and listen to others, we may not think alike, it is important to talk about it if we have a different vision, we don't laugh at other people's perspectives if they are different from ours
- Verbal assent
  - I will record our chat so I can remember what we said. I'm going to transcribe everything we said and remove your name, then I'm going to delete the recording. Then I will compare what all the students said to find similarities and differences in what was said to improve our project. I will also remove any information that you or I say that may identify you, so this includes your name. I will not share what you said to you teacher nor to your parents. Do you agree that I record and participate in this discussion? [if child says no, thank them for their time and explain that in that case we will not be doing the interview.]

2. Appreciation of the workshops

*We have done several workshops together in the last few weeks, can you tell me what we did? [reminder of workshops].*

- Can you start by telling me about your overall experience with the workshops?
- What were your challenges during the workshops?
- What were your favorite activities to do in the last few weeks?
  - Could you tell me about some of the aspects that made you like this activity?
- What activities did you like least?
  - Could you tell me about some of the aspects that made you not like this activity?
- In what situations do you think art activities can help at school?
  - How can they help?
- Would you like to continue doing art activities at school?
- What would you change about the way the activities are done?
- Were there any activities that you did not understand or where the instructions were not clear?
- Did you feel comfortable during the activities?
  - [No] What could have made you feel more comfortable?
  - [Yes] What made you feel comfortable?

3. Mental health

*Think about before we did the activities and now*

- Tell me about how things are at school
  - Do you see a difference between before we did the workshops and now?
- Do you feel that there is a difference in the space you have to express your ideas and perceptions?
  - If so, could you give me an example?
- Do you feel like you have more space to be free to be yourself?
  - What makes you feel freer to be yourself?
- Do you feel that there is a difference in your relationships with
  - (1) your friends (yes; ask for example)
  - (2) your parents (yes; ask for example)
  - (3) your teacher (yes; ask for example)
- Do you feel there is a difference in your feelings of competence?
  - If necessary, define competence as the ability to understand or do something (such as math, science, friendships, dance, etc.), so you feel capable of doing these things well.
- Do you feel that you are less discouraged than before?
- Why did you choose to participate in the workshops, what motivated you?
- If you think back to before we started the arts activities and now, what differences do you see in how you feel?
  - how you feel? the emotions you experience?
  - with your teacher? with your parents?
- Why do you think there have been these changes after the art activities?
- What was most helpful in the art activities?
  - Could you tell me more about this? How was it helpful?
- What other changes did you notice?
  - At home
  - At school
  - With your friends

4. Conclusions

- Do you have anything else to add about the activities?
- Do you have any questions for us?
- [Thank them for their participation]
