# Supplementary material for: Feasibility, acceptability, and perceived benefits of a creative arts intervention for elementary school children living with speech, language and communication disorders
Source: Front Child Adolesc Psychiatry. 2024 Jun 5;3:1322860. doi: 10.3389/frcha.2024.1322860 (PMC11748800; doi:10.3389/frcha.2024.1322860)
Supplement: Supplementary file 3 [file Table3.docx]

**Semi-structured Interview Guide for Teachers**

***Translated from French***

*Although the questions are presented in a separate order, they could be asked in a different order during the interview to allow participants to share their experiences freely.*

1. Introduction

- [Reiterate consent]
- The purpose of these interviews is to better understand your perception of the intervention and the impacts on your students.
- There is no right answer, we are looking to better understand was went well and what could be improved from your perspective.
- Could you tell me about the specific difficulties the children might have in your classroom?

2. Children's mental health

When thinking about the art activities we have done over the last 8 weeks:

- Have you seen an effect on the mental health of youth?
  - If so, could you give me some examples?
- How do you think the workshops have impacted the way youth express themselves to you and each other?
- How do you think art can help kids feel more:
  1. Competent
  2. Connected to others
  3. Autonomous
  - Do you think the activities we did allowed this?
- How did the activities impact their motivation in school?
- Have you noticed any other impacts on your students' mental health as a result of the workshops, either positive or negative?

3. Perception of the workshops

- How would you describe the level of engagement of the children in these workshops?
- Do you have any comments on how the activities went?
- Which workshops were most appreciated by you?
  - By the students?
- What are your recommendations for the program if it were to be repeated?
  - What might you change in the art activities?
  - What might you change overall?
- Do you have any comments on the procedures and feasibility of the program?
  - Were the material resources adequate?
  - Was the context suitable for the workshops (the class, the weather, the availability of the children, your availability, the number of students, etc.)?
- Instructions
  - How did you find the workshop instructions?
  - Did the number of participants, time and resources allow for meaningful participation? How could this have been improved?
- Would you recommend this program to colleagues in other classes?
- What specifically did you like about the program?
  - Less liked?

4. Thank you

- Thank you for welcoming us into your classroom and for your valuable participation.
